# Incidence and Associated Factors of Postoperative Pulmonary Complications after Abdominal Surgery in the Public Hospital, Addis Ababa, Ethiopia

**DOI:** 10.1155/2022/8223903

**Published:** 2022-07-06

**Authors:** Geresu Gebeyehu, Ashenafi Eshetu, Senait Aweke

**Affiliations:** ^1^Department of Anesthesia, College of Health Science, Addis Ababa University, Addis Ababa, Ethiopia; ^2^Department of Anesthesia, College of Medicine and Health Sciences, DebreBirhan University, DebreBirhan, Ethiopia

## Abstract

**Background:**

Postoperative pulmonary complications are a wide variety of disorders that affect normal respiratory functions, which in turn lead to morbidity and mortality. The extent to which it occurs is not yet studied in most clinical settings. This study assessed the incidence and risk factors of postoperative pulmonary complications in patients undergoing abdominal surgery under general anesthesia.

**Methods:**

A multicenter, prospective cross-sectional study was conducted at Menelik II, Tikur Anbessa Specialized, Zewditu Memorial, and Yekatit 12 Memorial hospitals after obtaining ethical clearance from each hospital. The study recruited a total of 287 participants using systematic random sampling. The data collection tool included sociodemographic, surgical, and anesthetic factors. Participants were followed for 7 days postoperatively, and any respiratory problems were recorded once identified. The collected data were entered and analyzed using SPSS version 26. Both bivariate and multivariate logistic regressions were used for analysis. A *p* value of <0.05 was considered statistically significant.

**Results:**

About 33% of the participants that underwent abdominal surgery developed postoperative pulmonary complications. Age > 65 years (AOR = 12.091, 95% CI = 3.310–44.169), duration of surgery >3 hours (AOR = 11.737, 95% CI = 3.621–38.039), preoperative oxygen saturation <94% (AOR = 10.671, 95% CI = 3.794–30.016), and postoperative serum albumin level <3.5 g/dl (*p* value <0.001) were associated with postoperative pulmonary complications significantly. *Conclusion and Recommendations.* The incidence of postoperative pulmonary complications after abdominal surgeries was high. Age >65years, duration of surgery >3 hours, preoperative SpO2% <94%, cigarette smoking, and postoperative serum albumin level <3.5 g/dl were factors strongly associated with postoperative pulmonary complications. We recommend special care for elderly patients, limit the surgical duration to less than 3 hours, treat the underlying cause of desaturation, and correct postoperative serum albumin to prevent the occurrence of postoperative pulmonary complications.

## 1. Introduction

A postoperative pulmonary complication is a broad term used to describe any adverse changes in the respiratory tract occurring after surgery and affecting the clinical course of the patients. The definitions for PPCs are derived from the European Society of Anesthesiology and the European Society of Intensive Care Medicine [1, 2].

Several works of the literature showed a wide variation in the occurrence of postoperative pulmonary complications ranging from 5 to 60% [1, 3–23]. Variation of definitions, preoperative tests to identify the associated risk, criteria used to diagnose, and diverse populations in different countries were the major contributory factors for developing postoperative pulmonary complications in a wide range [1]. A study conducted in Nigeria revealed that the rate of postoperative pulmonary complications was 52% and another study conducted in Zimbabwe and Ethiopia showed that 42.4% and 21.7% of patients developed postoperative pulmonary complications [4–8].

Many studies abroad have identified risk factors for postoperative pulmonary complications. The most identified risks are ASA > III, age ≥ 65 years, history of upper respiratory tract infection, use of general anesthesia, the presence of comorbidities, smoking, alcoholism, low socioeconomic status, desaturation less than 94%, serum albumin less than 3.5 gm/dl, intraoperative bleeding, hemoglobin less than 10 g/dl, intraoperative blood transfusion, prolonged surgery, postoperative mechanical ventilation, long hospital stay, cardiac surgery, history of previous respiratory diseases, poor postoperative pain control, use of neuromuscular blocking drugs, and early ambulation (3, 5–8, 9–15).

Knowledge about how PPCs occur, how to identify and treat postoperative pulmonary complications, and what factors contribute to it is lacking in most developing countries. This study hopefully aims in identifying those risk factors and developing strategies to reduce pulmonary complications.

## 2. Methods

### 2.1. Design, Duration, and Areas of Study

A prospective, multicenter, cross-sectional study was conducted at Menelik II, Tikur Anbessa Specialized, Zewditu Memorial, and Yekatit 12 hospitals, Addis Ababa, Ethiopia, from February 1 to April 30, 2020/21, after obtaining ethical approval from the ethical review board. The report of this article is made based on the revised STROCSS 2021 guideline.

### 2.2. Populations

#### 2.2.1. Source of Population

All patients who underwent elective and emergency abdominal surgery is in a selected governmental hospital, Addis Ababa, Ethiopia.

#### 2.2.2. Study Population

All surgical patients were scheduled for abdominal surgery during the study period and fulfilled inclusion criteria.

### 2.3. Eligibility Criteria

#### 2.3.1. Inclusion Criteria

All patients undergoing abdominal surgery and aged greater than 15 years were included in this study.

#### 2.3.2. Exclusion Criteria

The exclusion criteria were as follows:

  Pregnancy
  Procedures under regional anesthesia
  ICU patients

### 2.4. Study Variables

#### 2.4.1. Dependent Variables

The dependent variables include postoperative pulmonary complications: Yes/No.

#### 2.4.2. Independent Variables

Sociodemographic variables: age, sex, BMI, ASA status, and smoking status.

Preoperative factors: preoperative anemia, malignancy, renal failure, COPD, asthma, CHF, HTN, DM, and recent respiratory infection.

Anesthetic and surgical factors: type and duration of surgery, incision site, position, type of anesthesia, SpO2%, muscle relaxants, blood loss, transfusion, and intraoperative complications.

Postoperative factors: postoperative serum albumin, Chest Physiotherapy, pain, analgesic type, and ambulation.

### 2.5. Sample Size and Sampling Technique

#### 2.5.1. Sample Size Determination

The sample size was determined using the single population proportion method. A previously conducted study at Gondar University which reported the incidence of postoperative pulmonary complications as 21.7% [15] was used as a reference for sample size calculation by considering 95% and 5% margin of error.

Thus, it was computed as follows:

(1) $$\begin{array}{c} N=\frac{{\left(Z_{\alpha /2}\right)}^{2}\left(0.217\right)\left(0.783\right)}{{\left(0.05\right)}^{2}}=\frac{{\left(1.96\right)}^{2}\left(0.217\right)\left(0.783\right)}{{\left(0.05\right)}^{2}}=261. \end{array}$$

Adding a nonresponse rate of 10% gives the final sample size of 287.

#### 2.5.2. Sampling Technique

A systematic random sampling technique was assigned.

#### 2.5.3. Data Collection Techniques

Data were collected by using a pretested structured tool. Before the initiation of data collection, training was given to data collectors, and regular supervision was made throughout the collection process. The data collection starts during the preoperative period once informed consent was obtained from study participants. The data collection tool included preoperative demographic data, preoperative comorbidities, intraoperative anesthetic, surgical factors, and postoperative factors. The confidentiality was maintained throughout data collection procedures. All the above factors were recorded once observed by data collectors at any stage of data collection procedures. Patients were followed for seven days by data collectors during the postoperative period, and any new finding that suggested respiratory disorder was observed and confirmed by chest physicians after making full assessments.

### 2.6. Operational Definitions

Postoperative pulmonary complications: the occurrence of 2 or more of the signs and symptoms for at least 2 consecutive days, occurring within 7 days of surgery [12, 16–19]
  - Abnormal breath sounds: rhonchi/rales/decreased breath sounds
  - BAL/sputum culture-positive infective cause confirmed
  - Cough with sputum+ fever (*T* > 38)
  - Physicians/nurses' judgment of the respiratory cause
  - Respiratory rate >25/min
  - Saturation <90% room air, <94% with 100% oxygen for >2 hours
  - X-ray: consolidation/infiltrates/effusion new findings
Abdominal surgery: broadly covers surgical procedures that involve opening the abdomen
Postanesthesia care unit: A place where the patient is admitted after surgery and anesthesia to be given by professionals
Mobilization is defined as the ability to walk >10 m from the bed [20]

### 2.7. Data Processing, Analysis, and Interpretation

Data were checked for completeness code and entered into SPSS version 26. Descriptive statistics were computed, and the results were presented as frequency and percentage. A goodness fit test was conducted using the Hosmer and Lemeshow test. Both bivariate and multivariate logistic analyses were used to find out the associated factors. A variable with a *p* value ≤ of 0.2 from a bivariable was considered as a candidate for multivariate analysis. The strength of association was assessed using an odds ratio with a 95% confidence interval. The findings were presented using tables and graphs. The level of statistical significance was declared at a *p* value <0.05.

### 2.8. Data Quality Assurance

To assure the quality of data, training on the objectives and relevance of the study and brief orientations on the assessment tools were provided for data collectors. During data collection, all data were collected and properly filled in the prepared format. The supervisor controlled the data collector and checked for completeness daily after data collection. Daily data curation and cleanup were made by principal investigators.

### 2.9. Ethical Considerations

Ethical clearance and approval were obtained from the ethical review committee. An official support letter was written to each selected Addis Ababa governmental hospital and permission for data collection was sought from the responsible authorities. The purposes and the importance of the study were explained, and verbal as well as written informed consent was obtained from each participant. Confidentiality was ensured by avoiding personal identification on the questionnaire.

## 3. Results

### 3.1. Sociodemographic Variables

A total of 287 participants were recruited and completed the study. The findings for sociodemographic data are given in Table 1.

### 3.2. Perianesthetic and Surgical Factors

Various intraoperative anesthesia and surgery-related factors were assessed and the result is given in Table 2.

### 3.3. Postoperative Factors

The factors that contributed to postoperative pulmonary complications were analyzed and are given in Table 3.

### 3.4. Bivariate Analysis to Identify the Association of Independent Variables with Outcome Variables in Surgical Patients

Binary logistic regression analysis was conducted to identify the association of the outcome variable with each explanatory variable. Nine independent variables were included in the bivariate analysis. Exclude variables that do not fit for the final model using a *p* value >0.2 when multivariate analysis was performed. Four variables were selected for the multivariable model, and all of the variables were significant by using *p* value <0.05 (Table 4).

### 3.5. Multivariate Analysis of Factors Associated with Postoperative Pulmonary Complications in Surgical Patients

The results of the multivariate analysis are given in Table 5.

### 3.6. Overall Incidences of Postoperative Pulmonary Complications

**Figure 1:**
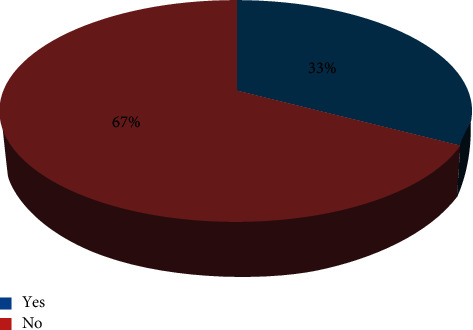
The incidence of postoperative pulmonary complications in patients who underwent abdominal surgery at public hospitals, Ethiopia.

The overall incidence of postoperative pulmonary complications was 33% and is shown in Figure 1.

### 3.7. Types of Pulmonary Problems Observed in Postsurgical Patients

**Figure 2:**
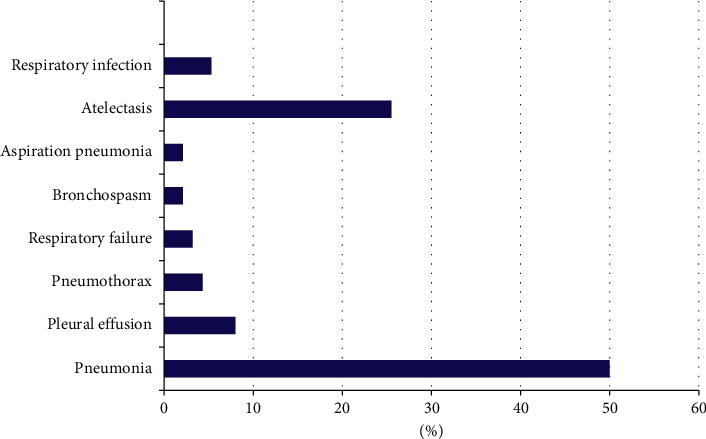
The distribution of postoperative pulmonary complications among patients who underwent abdominal surgery at public hospitals, Ethiopia.

Among different types of PPCs, pneumonia (50%) and atelectasis (24) were the commonest ones. The graph shows different types of disorders affecting the respiratory system, as shown in Figure 2.

## 4. Discussion

This study revealed the incidence of postoperative pulmonary complications after abdominal surgeries was 33%. This finding is higher than that of the report of the study conducted at Gondar University Hospital in 2015 [15]. The possible explanation for this could be a larger sample size used in their study, and interobserver variability could have the potential to affect the finding. However, this finding was comparable with the results of the observational analytical study conducted in India by Sinouvassan et al. which came out with an incidence of 34% [21]. Our study's finding was also in line with a retrospective study conducted in Turkey by Diken et al. which showed an incidence of 32.6% [22]. This might be due to the similarity in criteria used to diagnose postoperative pulmonary complications or postoperative follow-up periods.

Meanwhile, the finding of this study was lower than that of the study conducted in Zimbabwe by Tadyanemhandu et al. , Harare, which revealed that 42.4% of study subjects developed postoperative pulmonary complications [3]. Moreover, an observational cohort study conducted in an Australian tertiary hospital by Haines et al. and Parry et al. revealed incidences of 39% and 42%, respectively [23,24]. The differences in the study design may have contributed to this discrepancy in the incidences.

Participants who had postoperative serum albumin levels <3.5 g/ were 23 times more likely to develop postoperative pulmonary complications (*p* ≤ 0.001). The possible explanation would be albumin is necessarily indicating the nutritional status of the patients, is important for muscle strength, and also promotes the wound healing process. The serum albumin level indicates the nutrition status and associated weakness of the expiratory muscles, decreased chest wall expansion, and an increased incidence of pulmonary complications in patients [25].

Age greater than 65 years old was 12 times riskier of postoperative pulmonary complications (*p* ≤ 0.001, AOR: 12.09, 95% CI: 3.31–44.1). The likely cause could be aging was related to increased cardiorespiratory comorbidities which can be aggravated during surgery and anesthesia [13, 26].

Surgical duration longer than 3 hours would be 11 times more likely to have postoperative pulmonary complications (*p* ≤ 0.001, AOR: 11.7, 95% CI: 3.62–38.03). This finding is in line with different pieces of the literature studied by different researchers abroad [3, 12, 15]. The possible explanation may be the long duration of surgery was associated with an altered physiological response of metabolic activities [13, 25].

Study participants who had preoperative oxygen saturation <94% were 10 times riskier for postoperative pulmonary complications (*p* ≤ 0.001, AOR: 10.67 95% CI: 3.794–30.016). Our result is in line with research done in China by Jin et al. and in Spain by Canet et al. [7, 13].

### 4.1. Strength and Limitations of the Study

The study identified that pulmonary complications can affect a significant number of surgical patients during the postoperative period. It also identified major associated factors that had the potential to contribute to the occurrence of postoperative pulmonary complications. The limitation of the study was that there can be another associated factor, which may contribute to the problem and interobserver variability in diagnosing postoperative pulmonary complications.

## 5. Conclusion and Recommendations

The postoperative complication was found to occur significantly in patients undergoing both upper and lower abdominal surgeries. The study revealed age >65 years, duration of surgery >3 hours, SpO_2_% <94%, and the postoperative serum albumin level <3.5 g/dl were factors strongly associated with postoperative pulmonary complications. We recommend the healthcare workers to give a deep insight into the identified factors and take necessary precautions while caring for the patients. A future study with a large sample size and large varieties of surgical specialties is recommended.

## Figures and Tables

**Table 1:** The sociodemographic characteristics of patients who underwent abdominal surgery in public hospitals, Ethiopia.

Variables		Frequency (*n*)	Percentage (%)
Age	15–47	166	57.8
	48–63	55	19.2
	≥64	66	23

Gender	Female	143	49.8
	Male	144	50.2

BMI	<18.5	36	12.5
	18.5–24.9	220	76.7
	25–34.9	30	10.5
	>35	1	0.3

ASA status	ASA I	154	53.7
	ASA II	133	46.3

Type of anesthesia	General	249	86.8
	Regional	38	13.2

Type of operation	Elective	117	40.8
	Emergency	170	59.2

Alcoholic	Yes	46	16
	No	241	84

Preoperative anemia	Yes	33	11.5
	No	254	88.5

Preoperative saturation	<94%	99	34.5
	≥94%	188	65.5

Malignancy	Yes	34	11.8
	No	253	88.2

Hypertension	Yes	29	10.1
	No	258	89.9

Diabetes mellitus	Yes	21	7.3
	No	266	92.7

The values are expressed in numbers and percentages. Ages are expressed in years.

**Table 2:** Intraoperative anesthesia and surgical factors in patients who underwent abdominal surgery at public hospitals, Ethiopia.

Variables		Frequency (*n*)	Percentages (%)
Surgical site	Upper abdominal	169	58.9
	Lower abdominal	46	16
	Both	72	25.1

Incision type	Vertical	146	50.9
	Horizontal	30	10.5
	Transverse	70	24.4
	Subcostal	41	14.3

Muscle relaxant	Short-acting	17	5.9
	Intermediate	216	75.3
	Long-acting	8	2.8
	None	46	16

Surgical position	Supine	268	93.4
	Lateral	5	1.7
	Lithotomy	14	4.9

Blood transfusion	Yes	36	12.5
	No	251	87.5

Intraoperative blood loss	≤500 ml	218	76
	>500 ml	69	24

The values are expressed in numbers and percentages.

**Table 3:** The postoperative factors in patients who underwent abdominal surgery at public hospitals, Ethiopia.

Variables		Frequency (*n*)	Percentage (%)
Postoperative albumin	<3.5	100	34.8
	≥3.5	187	65.2

NRS pain score	None	24	8.4
	Mild	138	48.1
	Moderate	98	34.1
	Severe	27	9.4

Postoperative mobility	≤24 h	152	53
	>24 h	135	47

The values are expressed in numbers and percentages.

**Table 4:** Bivariate analysis to assess the association of variables with postoperative pulmonary complications in surgical patients in public hospitals, Ethiopia.

Variables	Category	Yes (%)	No (%)	COR, 95% CI	*P* value
Age	15–47	25 (26.6%)	141 (73.1%)	1	
	48–63	15 (16%)	40 (20.7%)	0.735 (0.136–3.971)	0.720
	≥64	54 (57.4%)	12 (6.2%)	0.078 (0.011–0.552)	0.011^*∗*^

Surgical procedure	Upper abdominal	51 (54.3%)	118 (61.1%)	4.033 (0.119–137.217)	0.438
	Lower abdominal				
	Both				
		12 (12.8%)	34 (17.6%)	1	
				
		31 (33%)	41 (21.2%)	6.463 (0.125–334.303)	0.354

Type of anesthesia	General	83 (88.3%)	166 (86%)	0.177 (0.005–6.167)	0.339
	Regional	11 (11.7%)	27 (14%)	1	

Duration of surgery	<2 h	13 (13.8%)	126 (65.3%)	1	
	2-3 h	12 (12.8%)	51 (26.4%)	2.003 (0.286–14.026)	0.484
	>3 h	69 (73.4%)	16 (8.3%)	0.064 (0.011–0.381)	0.003^*∗*^

Type of operation	Elective	40 (42.6%)	77 (39.9%)	1	
	Emergency	54 (57.4%)	116 (60.1%)	0.574 (0.117–2.809)	0.493

Smoking status	Never smoker	42 (44.7%)	167 (86.5%)	1	
	Former smoker	9 (9.6%)	13 (6.7%)	1.005 (0.097–10.354)	0.997
	Current smoker				
		43 (45.7%)	13 (6.7%)	0.453 (0.069–2.992)	0.411

Preoperative saturation	<94%	77 (81.9%)	22 (11.4%)	0.257 (0.057–1.164)	0.078^*∗*^
	≥94%	17 (18.1%)	171 (88.6%)	1	

Postoperative serum albumin	<3.5	79 (84%)	21 (10.9%)	0.067 (0.014–0.322)	0.067^*∗*^
	≥3.5	15 (16%)	172 (89.1%)	1	

Pain score	None	0	24 (12.4%)	1	
	Mild	4 (4.3%)	134 (69.4%)	0.000	
	Moderate	63 (67%)	35 (18.1%)	0.000	
	Severe	27 (28.7%)	0	0.000	

COR, crude odds ratio; 1, reference. ^∗^The variables that were candidate for multivariate analysis.

**Table 5:** Factors associated with postoperative pulmonary complication in abdominal surgical patients at public hospitals, Ethiopia.

Variable		PPCs		COR (95% CI)	AOR (95%)	*P* value
		Yes	No			
Age	15–47	25 (26.6%)	141 (73.1%)	1	1	0.593
	48–63	15 (16%)	40 (20.7%)	0.735 (0.136–3.971)	1.399 (0.409–4.783)	
	≥64	54 (57.4%)	12 (6.2%)	0.078 (0.011–0.552)	12.091 (3.310–44.169)	≤0.001^*∗∗*^

Duration of surgery	<2 h	13 (13.8%)	126 (65.3%)	1	1	0.956
	2-3 h	12 (12.8%)	51 (26.4%)	2.003 (0.286–14.026)	1.039 (0.267–4.045)	
	>3 h	69 (73.4%)	16 (8.3%)	0.064 (0.011–0.381)	11.737 (3.621–38.039)	≤0.001^*∗∗*^

Preoperative saturation	<94%	77 (81.9%)	22 (11.4%)	0.257 (0.057–1.164)	10.671 (3.794–30.016)	≤0.001^*∗∗*^
	≥94%	17 (18.1%)	171 (88.6%)	1	1	

Postoperative serum albumin	<3.5	79 (84%)	21 (10.9%)	0.067 (0.014–0.322)	23.407 (7.956–68.865)	≤0.001^*∗∗*^
	≥3.5	15 (16%)	172 (89.1%)	1	1	

1, reference; COR, crude odds ratio; AOR, adjusted odds ratio. ^*∗∗*^Statistically significant.

## Data Availability

The data used to support this study are available from the corresponding author upon request.

## Abbreviations

ASA: American Society of Anesthesiologists
BMI: Body mass index
EPCO: European perioperative clinical outcome
ICU: Intensive care unit
LOS: Length of hospital stay
NRS: Numerical rating scale
PPCs: Postoperative pulmonary complications
SpO_2_%: Arterial oxyhemoglobin saturation
SPSS: Statistical Package of the Social Sciences
TASH: Tikur Anbessa Specialized Hospital.
